# Microbial Responses to Simulated Salinization and Desalinization in the Sediments of the Qinghai–Tibetan Lakes

**DOI:** 10.3389/fmicb.2020.01772

**Published:** 2020-08-07

**Authors:** Jianrong Huang, Jian Yang, Hongchen Jiang, Geng Wu, Wen Liu, Beichen Wang, Haiyi Xiao, Jibin Han

**Affiliations:** ^1^State Key Laboratory of Biogeology and Environmental Geology, China University of Geosciences, Wuhan, China; ^2^State Key Laboratory of Desert and Oasis Ecology, Xinjiang Institute of Ecology and Geography, Urumqi, China; ^3^Faculty of Materials Science and Chemistry, China University of Geosciences, Wuhan, China; ^4^Key Laboratory of Salt Lake Geology and Environment of Qinghai Province, Qinghai Institute of Salt Lakes, Northwest Institute of Eco-Environment and Resources, Chinese Academy of Sciences, Xining, China

**Keywords:** microbial community, salinization, desalinization, lake sediments, salinity

## Abstract

Uncovering microbial response to salinization or desalinization is of great importance to understanding of the influence of global climate change on lacustrine microbial ecology. In this study, to simulate salinization and desalinization, sediments from Erhai Lake (salinity 0.3–0.8 g/L) and Chaka Lake (salinity 299.3–350.7 g/L) on the Qinghai–Tibetan Plateau were transplanted into different lakes with a range of salinity of 0.3–299.3 g/L, followed by *in situ* incubation for 50 days and subsequent geochemical and microbial analyses. Desalinization was faster than salinization in the transplanted sediments. The salinity of the transplanted sediment increased and decreased in the salinization and desalinization simulation experiments, respectively. The TOC contents of the transplanted sediments were lower than that of their undisturbed counterparts in the salinization experiments, whereas they had a strong negative linear relationship with salinity in the desalinization experiments. Microbial diversity decreased in response to salinization and desalinization, and microbial community dissimilarity significantly (*P* < 0.01) increased with salinity differences between the transplanted sediments and their undisturbed counterparts. Microbial groups belonging to *Gammaproteobacteria* and *Actinobacteria* became abundant in salinization whereas *Bacteroidetes* and *Chloroflexi* became dominant in desalinization. Among the predicted microbial functions, hydrogenotrophic methanogenesis, methanogenesis through CO_2_ reduction with H_2_, nitrate/nitrogen respiration, and nitrification increased in salinization; in desalinization, enhancement was observed for respiration of sulfur compounds, sulfate respiration, sulfur respiration, thiosulfate respiration, hydrocarbon degradation, chemoheterotrophy, and fermentation, whereas depressing was found for aerobic ammonia oxidation, nitrate/nitrogen respiration, nitrification, nitrite respiration, manganese oxidation, aerobic chemoheterotrophy, and phototrophy. Such microbial variations could be explained by changes of transplantation, salinity, and covarying variables. In summary, salinization and desalinization had profound influence on the geochemistry, microbial community, and function in lakes.

## Introduction

Lakes are distributed globally and respond sensitively to regional and/or global environmental changes (Adrian et al., 2009; O’Reilly et al., 2015). Lake salinization and desalination are growing environmental problems caused by both human activities (Dugan et al., 2017) and climate changes (Fedotov et al., 2013). Continuous anthropogenic perturbations and climate changes (e.g., drying, humidification) often lead to lake surface area shrinkage or enlargement, resulting in salinization (i.e., salinity increase) (Micklin, 2007; Guo et al., 2015) or desalination (i.e., salinity decrease) (Schulz, 2000; Fedotov et al., 2013), respectively. Long-term salinization and desalination constantly change the salinity and other physiochemical variables in lakes. Salinity significantly influences microbial diversity, community structure, and metabolic activities (Jiang et al., 2006; Wang et al., 2011; Yang et al., 2019). However, little is known about how salinization and desalinization affect microbial community compositions and their metabolic functions in lakes.

Previous investigations about salinity effect on microbial community compositions have largely been limited to estuaries with a continuous salinity gradient (Bouvier and del Giorgio, 2002; Herlemann et al., 2011) and to saline and hypersaline lakes with different salinity (Yang et al., 2019, and references therein). However, these previous studies rarely involve the response of one specific microbial community to a full range of salinity (i.e., from freshwater to salt saturation) and determine the difference between effects of salinization and desalinization on one specific microbial community. In addition, lake communities consist of various microbial functional groups, such as methanogenesis and sulfate reduction, and they are actively involved in element cycling in lakes (Kosolapov et al., 2003; Sorokin et al., 2015; Fuchs et al., 2016; Wörner and Pester, 2019). Previous investigations indicated that methanogenesis was more common in freshwater environments than in saline environments, whereas an opposite trend was observed for sulfate reduction (Kulp et al., 2007; Liu et al., 2016; Dang et al., 2019). Accordingly, studying dynamics in microbial function successions and their response to salinization or desalinization is of great importance to understanding of microbial functions in element cycling in lacustrine ecosystems. Furthermore, microbes in lakes with different salinity may possess distinct community compositions and metabolic functions; sediment mineralogy also differs significantly among lakes with different salinity, so as to provide different sediment matrices (ecological niches) for microbes therein (Yang et al., 2020). So it is reasonable to speculate that microbes from different lakes may respond distinctly to salinity variation caused by salinization or desalinization. Therefore, in order to study the response of sediment microbial community to salinization or desalinization, it is necessary to perform simulation experiments by transplanting sediment from one lake to other environments with different salinity.

Transplanting experiments provides a valid field experiment strategy for studying microbial response to salinity variation (Gasol et al., 2002; Reed and Martiny, 2007). Transplanting experiments can be established by caging sediment into semipermeable membrane, through which microbes cannot exchange between inside and outside of the cages but inorganic ions and nutrition can exchange freely. Using this method, one previous study observed that microbial community structures and functions in tidal freshwater wetlands were changed under elevated salinity (Dang, 2016). Likewise, two transplanting experiments were performed between low- and high-salinity locations in the Nonesuch River estuary, and it turned out that salinity variations altered the abiotic conditions (e.g., salinity, sulfate, pH, and ammonium), and microbial community compositions and related functional processes in the transplanted sediments (Reed and Martiny, 2013). However, these previous transplanting studies seldom involve changes of microbial communities and functions in sediments with a salinity range higher than seawater salinity (∼35 g/L).

The Qinghai–Tibetan Plateau (QTP) hosts thousands of lakes (>1 km^2^) (Zheng, 1997). These lakes possess a large range of salinity (from 0.1 to 426.3 g/L) and pH (5.4–10.2). Lake sediment records in the QTP indicated that during the past 730,000 years this region has experienced multiple drought and humid climate cycles, which may have led to lake salinization and desalination (Huang and Chen, 1990). In addition, the QTP is very sensitive to global and local environmental changes (e.g., global warming), and most of the QTP lakes are undergoing desalinization, which is evidenced by the continuous water level increase and lake surface area expansion (Zhang G. et al., 2017, 2019; Li et al., 2019). Hence, the QTP lakes are ideal for examining effect of salinization and desalinization on microbial community and functions. Therefore, in order to fill the above knowledge gaps, transplanting experiments were established by caging sediments from two QTP lakes (one freshwater lake and the other salt-saturation lake), followed by incubation into different lakes with a full range of salinity (from freshwater to salt saturation) and subsequent geochemical and microbial analyses.

## Materials and Methods

### Site Description

Five lakes (Erhai Lake, Tuosu Lake, Gahai Lake2, Xiaochaidan Lake, and Chaka Lake) with a full range of salinity (from freshwater to salt saturation) on the northern Qinghai–Tibetan Plateau (36°33′–37°27′ N, 95°30′–100°43′ E, 2800 m above sea level) were selected in this study (Supplementary Figure S1). The study region is characteristic of typical continental arid and semi-arid climate with mean annual precipitation of 350 mm. Erhai Lake (EHL) (salinity 0.3–0.8 g/L, freshwater) has a surface area of 5 km^2^ and maximum water depth of 2.0 m (Jiang et al., 2009). Tuosu Lake (TSL) (salinity 25.3–25.4 g/L, saline) has a surface area of 165.9 km^2^ and maximum water depth of 23.6 m. Gahai Lake2 (GHL2) (salinity 29.3–33.5 g/L, saline) has a surface area of 35 km^2^ and maximum water depth of 15.0 m. Xiaochaidan Lake (XCDL) (salinity 75.2–78.1 g/L, hypersaline) covers an area of 71.5 km^2^ with the maximum water depth of 1.0 m. Chaka Lake (CKL) is a shallow lake (salinity 299.3–350.7 g/L, salt), with a surface area of 105 km^2^ and an average water depth of 20–30 cm (Jiang et al., 2007). The spatial distances among these five lakes ranged from 200 to 600 km.

### Experimental Design and Sediment Sampling

**FIGURE 1:**
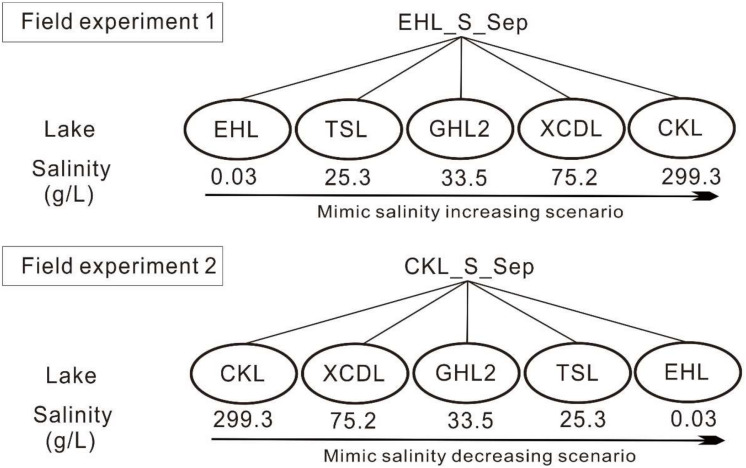
Experimental design. Experiment 1 was to simulate increasing salinity scenario (salinization); the freshwater sediments (EHL_S_Sep) were transplanted into EHL, TSL, GHL2, XCDL, and CKL, respectively. Experiment 2 was to simulate decreasing salinity scenario (desalinization): the salt lake sediments (CKL_S_Sep) were transplanted into CKL, XCDL, GHL2, TSL, and EHL, respectively.

In September 2013, surface sediment (with a mean depth of 1–5 cm) samples were collected in triplicate (the distance is about 200–300 m among the triplicate sampling sites) from the five investigated lakes by using a grab-bucket collection sampler (XDB0201, New Landmark, China). The triplicate sediments from each lake were mixed into a unique representative analytical sample (Take EHL_S_Sep as an example, suffixed with -S indicates sediments; and Sep indicates the sampling time in September). Then the sediments from EHL (representing the freshwater lake end) and CKL (representing the salt lake end) were split into two technical replicates (suffixed with “1” or “2” after “-E1” or “-E2”; E1 and E2 indicated “Experiment 1” and “Experiment 2,” respectively. i.e., EHL_E1_1) and were separately collected into dialysis bags (∼20 cm long and 10 cm in diameter) (Spectrum Laboratories, CA, United States) to establish transplanting cages as described previously (Reed and Martiny, 2013). “Experiment 1” and “Experiment 2” simulated the scenarios of salinization and desalinization in lakes, respectively. The established sediment cages were sealed and subsequently were incubated onto the sediment surface of the target lakes (Figure 1). In the salinization simulation experiment, the caged freshwater sediments (EHL_S_Sep) were transplanted into EHL, TSL, GHL2, XCDL, and CKL, respectively, and in the desalinization simulation experiment, the caged salt lake sediments (CKL_S_Sep) were transplanted into CKL, XCDL, GHL2, TSL, and EHL, respectively (Figure 1).

After 50-day incubation, all the 20 sediment cages were recovered from each lake in November 2013. Upon retrieval, approximately 5 g of sediments in the center of each dialysis bag was aseptically collected into Eppendorf tubes. For comparison, the surface sediment sample of the five incubating lakes were also collected in an identical way in September (beginning of the incubation) and November (end of the incubation). These lakes were suffixed with “Sep” and “Nov,” indicating the sampling time. Note that one transplanted sediment sample replicate (XCDL_E1_1) was lost. A total of 29 samples (10 lake sediment samples collected in September and November, 19 transplanted samples) were obtained. All the collected samples for geochemical and microbial analyses were stored in dry ice during transportation and in a −80°C freezer in laboratory until further analysis.

### Geochemical Analyses

In laboratory, sediment porewater was obtained by centrifugation at 7200 × *g* for 30 min. The sediment pore water was used for pH determination by a digital soil pH meter (UB-7; Denver Inc., Germany). Major cation and anion concentrations (e.g., K^+^, Na^+^, Ca^2+^, Mg^2+^, Cl^–^, SO_4_^2–^) of sediment porewater were measured by using ion chromatography (Dionex DX-600, United States). Salinity was calculated as the sum of the concentrations of the measured ions (K^+^, Na^+^, Ca^2+^, Mg^2+^, Li^+^, Cl^–^, SO_4_^2–^, NO_2_^–^, NO_3_^–^, and Br^–^) in the unit of grams per liter. The total organic carbon (TOC) content in the sediment was measured using a multi N/C 2100S analyzer (Analytik Jena, Germany).

### DNA Extraction, PCR Amplification, and Pyrosequencing

Total DNA was extracted from all the collected sediments by using the FastDNA Spin Kit for Soil (MP Biomedical, OH, United States) according to the manufacturer’s instructions. The universal V4 hypervariable region of the 16S rRNA genes was PCR amplified with the use of a set of bar-coded primers of 515F (5′-GTGYCAGCMGCCGCGGTAA-3′)/806R (5′-CCGGACTACHVGGGTWTCTAAT-3′). PCR conditions followed a previous study (Tamaki et al., 2011). Briefly, a unique 12 bp barcode sequence was added to the reverse primer (806R) to differentiate among samples. Triplicate PCR reactions for each sample were conducted. PCR products were purified using a DNA gel extraction kit (Axygen, Union City, CA, United States). Barcoded amplicons (∼400 bp) from each sample were pooled with equimolar concentrations and then were sequenced by using an Illumina-Miseq platform (Caporaso et al., 2012).

### Processing of Illumina Sequencing Data

Raw sequences were processed in the QIIME2 (Caporaso et al., 2010) software version 2018.6 following the recommended tutorials^1^. Briefly, raw sequences were quality trimmed and assigned to samples based on unique sample tags. Sequences containing ambiguous nucleotide bases and mismatches during filtering were discarded. Chimera checking was performed using UCHIME (Edgar et al., 2011). The effective sequences were clustered into amplicon sequence variants (ASVs) at the 100% identity level (Callahan et al., 2017) in the resulting feature table. To avoid possible biases, ASVs containing singletons were removed from the feature table for downstream analysis. The taxonomic of representative ASV sequences were determined using the RDP classifier at a confidence level of 80% (Wang et al., 2007). To compare all of the samples at the same level of sampling effort, 5744 sequences (corresponding to the smallest sequencing effort among the samples) were randomly selected before the alpha- and beta-diversity analyses. The α-diversity indices including ASVs richness, Simpson, Shannon, and Equitability indices were calculated using the “vegan” package.

### Statistical Analyses

Subsequent analyses were performed in the R program^2^ using various packages for analysis of ecological data. To avoid possible biases, ASVs present less than twice in the two biological replicates were removed from the feature table before further analysis. Overall compositions changes of microbial community were evaluated by principal coordination analysis (PCoA) in the “vegan” package based on Bray–Curtis dissimilarity. One-way and two-way PERMANOVA (permutations = 999, method = “bray”) were performed, using the “adonis” function in “vegan” package (Oksanen et al., 2015), to test the effects of transplantation, salinity, and their interaction on community composition. Mantel test was applied to assess the relationships between the microbial community and the measured environmental variables using the “vegan” package. Linear regression analyses were used to assess the correlation between salinity and TOC/alpha diversity/relative abundances of potential microbial functions by using “basicTrendline” package. Linear regression model was also employed to examine the correlation between salinity difference and microbial community dissimilarity based on Bray–Curtis distance in sediment transplanting experiments. Venn was performed using “VennDiagram” in the R package (Chen and Boutros, 2011). Functional Annotation of Prokaryotic Taxa (FAPROTAX) was used to predict established metabolic prediction from the valid 16S rRNA sequences based on the characterized strains (Louca et al., 2016). The obtained ASV tables were compared with putative functional tables based on taxa and their functional annotations in the FAPROTAX database.

### Nucleotide Sequence Accession Numbers

The raw reads were deposited into the NCBI Sequence Read Archive database (accession nos. SRR1303664–SRR1303692) under the BioProject ID PRJNA248705.

## Results

### Sediment Characteristics and Porewater Geochemistry

The geochemical characteristics of the lake sediments were summarized in Supplementary Table S1. The sediments of the five incubating lakes covered a wide range of salinity from freshwater to salt saturation (from 0.3 to 350.7 g/L). TOC contents of these lake sediments ranged from 0.2 to 3.7% with pH 7.3–8.5. Major ions of the sampled lake sediments varied widely (Supplementary Table S1). For example, Na^+^ concentrations ranged from 62.0 to 55,366.4 mg/L; Cl^–^ concentrations ranged from 113.2 to 208,324.4 mg/L; Mg^2+^ concentrations ranged from 18.8 to 487.6 mg/L; SO_4_^2–^ were 46.8–45,936.7 mg/L; Ca^2+^ were 27.4–37,291.8 mg/L; and K^+^ were 26.3–6210.1 mg/L. Such large variations in sediment geochemistry characteristics are beneficial to assess the impact of salinization and desalinization on the microbial distribution in lacustrine ecosystem.

### Geochemistry Variations After Sediment Transplantation

**FIGURE 2:**
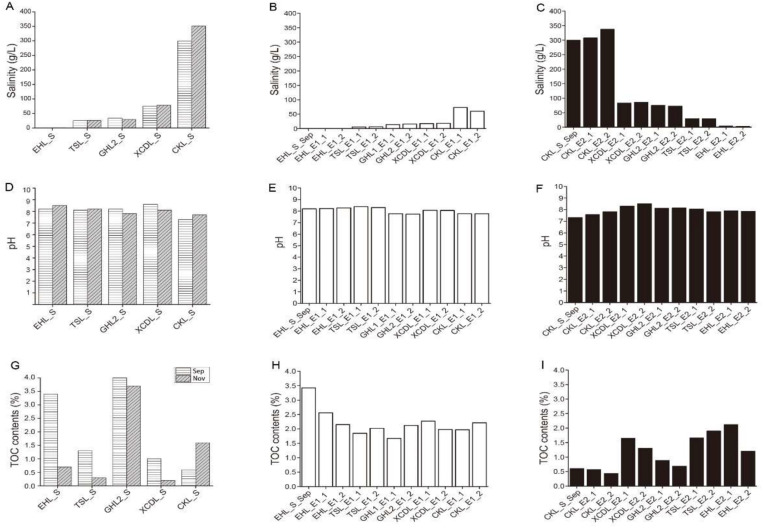
Means of **(A–C)** salinity, **(D–F)** pH and **(G–I)** TOC contents in the lake and transplanted sediments.

Geochemistry of the caged sediments changed after transplantation (Figure 2). In experiment 1 (simulating salinization), salinity of the transplanted sediments increased from 0.3 to 73.4 g/L (maximum), but was not in equilibrium with that of the incubating lakes (lower than that of the incubating lakes) (Figures 2A,B); pH of the transplanted sediments was similar to that of the incubating lakes (Figures 2D,E) and the undisturbed EHL sediments (EHL_S_Sep, Figure 2E), whereas the TOC contents of the transplanted sediments were lower than that of the undisturbed EHL sediments (EHL_S_Sep, Figure 2H). In contrast, in experiment 2 (simulating desalinization), salinity of the transplanted sediment decreased from 299.3 to 3.2 g/L (minimum), and was almost in equilibrium with that of the incubating lakes (Figures 2A,C). Similar to experiment 1, pH of the transplanted sediment approached to that of the incubating lakes (Figures 2D,F) and the undisturbed CKL sediments (Figure 2F), whereas the TOC contents of the transplanted sediments were relatively higher than that of the undisturbed CKL sediment (CKL_S_Sep, Figure 2I). Linear regression analysis showed that salinity had a strong negative linear relationship (*R*^2^ = 0.605, *P* < 0.01) with the TOC contents of the transplanted sediments in experiment 2 (simulating desalinization), whereas no significant correlation (*R*^2^ = 0.077, *P* > 0.1) was observed between salinity and the TOC contents of the transplanted sediments in experiment 1 (simulating salinization) (Supplementary Figure S2).

### Microbial Alpha Diversity in the Lake Sediments and Transplanted Sediments

**FIGURE 3:**
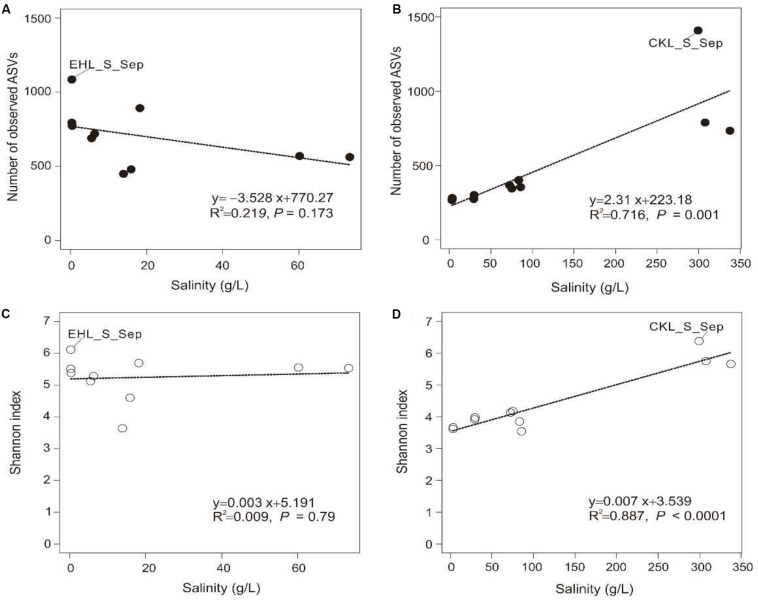
Relationship between salinity and alpha diversity (observed ASVs, Shannon diversity). **(A,C)** Relationship between salinity and observed ASVs, Shannon index in experiment 1 (simulating salinization), respectively. **(B,D)** Relationship between salinity and observed ASVs, Shannon index in experiment 2 (simulating desalinization), respectively. Linear regression is shown in the bottom right corner.

A total of 328,960 high-quality reads were generated with an average of 8435 sequence reads per sample. The obtained high-quality sequence reads could be clustered into 7153 ASVs at 100% identity. Alpha diversity indices of the studied samples are summarized in Supplementary Table S2. Among the lake sediments, the observed ASVs and Shannon index were 522–1409 and 4.69–6.38, respectively (Supplementary Table S2). Microbial diversity in the transplanted sediments was lower than that in their undisturbed counterparts and generally decreased in both of the salinization and desalinization experiments. Furthermore, there was no significant correlation between salinity and the microbial diversity (observed ASVs or Shannon index) in experiment 1 (simulating salinization) (Figures 3A,C), whereas a significant (*P* < 0.001) correlation was observed between salinity and the microbial diversity (Figures 3B,D) in experiment 2 (simulating desalinization).

### Overall Microbial Communities in the Lake Sediments and Transplanted Sediments

**FIGURE 4:**
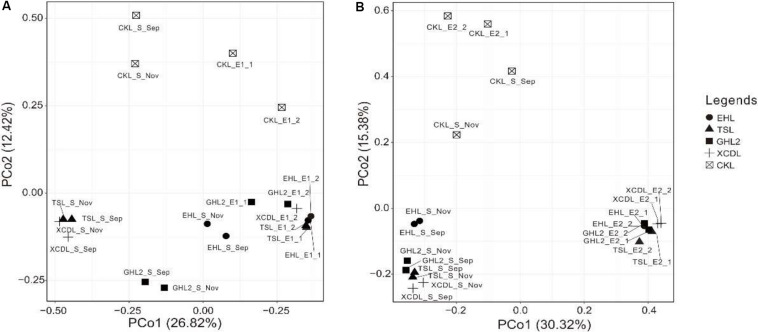
PCoA plots (based on Bray–Curtis dissimilarity matrix) showing relative compositional differences between the lake sediments and transplanted sediments in experiments 1 and 2 (**A,B**: simulating salinization and desalinization, respectively). Values on PCoA axes indicate the percentages of total variation explained by each axis.

The microbial communities in the transplanted sediments were clearly separated from those in the sediments of the incubating lakes in both of the salinization and desalinization experiments (Figure 4). Furthermore, the microbial communities in the transplanted sediments retrieved from EHL (i.e., EHL_E1, in experiment 1) and CKL (i.e., CKL_E2, in experiment 2) were also different from their undisturbed counterparts. In addition to the clear effect of transplantation on transplanted microbial communities, there was an apparent impact of salinity on transplanted communities such as CKL (with highest salinity) and other four transplanted sediments in both of the salinization and desalinization experiments, which was manifest on the PCoA results (Figure 4). In addition, for the incubating lakes (except CKL), microbial dissimilarity between the sediments collected in September and November was less than that among lakes (Figure 4).

### Effects of Transplantation, Salinity, and Covarying Variables on the Microbial Communities in the Transplanted Sediments

**FIGURE 5:**
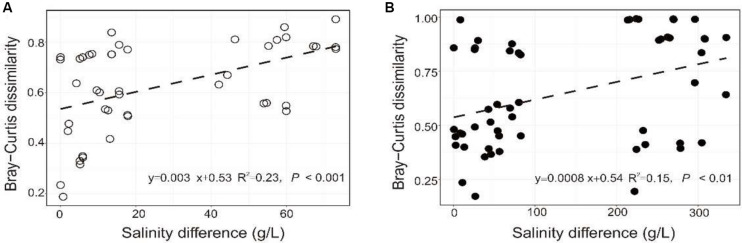
Relationship between the microbial community difference of the experiment 1 (**A**: simulating salinization) and experiment 2 (**B**: simulating desalinization) and sedimentary salinity difference.

The PERMANOVA test showed that the microbial dissimilarity between the transplanted and undisturbed sediments was significant (*P* < 0.01) with transplantation and salinity (Table 1). In comparison, salinity showed smaller correlation coefficients than transplantation in both the salinization and desalinization simulation experiments. Mantel test showed that the microbial community compositions in the transplanted sediments were significantly correlated with salinity and major ions including K^+^, Na^+^, Mg^2+^, Cl^–^, SO_4_^2–^, and NO_2_^–^ in experiment 1 (simulating salinization), and with salinity, TOC contents and major ions including K^+^, Na^+^, Mg^2+^, Ca^2+^, Li^+^, Cl^–^, SO_4_^2–^, and NO_2_^–^ in experiment 2 (simulating desalinization) (Table 2). Meanwhile, the microbial dissimilarity (based on Bray–Curtis dissimilarity) showed significant positive linear correlations (E1: *R*^2^ = 0.23, *P* < 0.001; E2: *R*^2^ = 0.15, *P* < 0.01) with the salinity difference between the transplanted sediments and their undisturbed counterparts (Figure 5).

**TABLE 1:** Influences of transplantation and salinity on the microbial dissimilarity (based on Bray–Curtis distance) between the transplanted and undisturbed sediments as indicated by permutational multivariate analysis of variance (PERMANOVAS) with ADONIS function (permutation: 999).

	**Transplantation**		**Salinity**		**Transplantation and salinity**	
	***R*^2^**	***P***	***R*^2^**	***P***	***R*^2^**	***P***
Experiment 1 (salinity increases)	0.23	<0.01	0.13	<0.01	0.40	<0.01
Experiment 2 (salinity decreases)	0.24	<0.01	0.14	<0.01	0.48	<0.01

**TABLE 2:** Mantel test showing Spearman’s correlation (r) between the microbial community structures and environmental variables of the transplanted sediments (permutation: 9999).

**Environmental variables**	**Experiment 1 (salinity increases)**		**Experiment 2 (salinity decreases)**	
		
	***r***	***P***	***r***	***P***
Salinity	+0.556	<0.01	+0.852	<0.001
pH	+0.204	0.061	+0.221	0.122
TOC	+0.274	0.159	+0.383	<0.05
K^+^	+0.581	<0.01	+0.900	<0.001
Na^+^	+0.567	<0.01	+0.822	<0.001
Mg^2+^	+0.526	<0.05	+0.916	<0.001
Ca^2+^	−0.038	0.478	+0.472	<0.05
Li^+^	+0.436	0.198	+0.801	<0.01
Cl^–^	+0.543	<0.01	+0.846	<0.001
SO_4_^2–^	+0.489	0.022	+0.818	<0.001
NO_2_^–^	+0.341	<0.05	+0.652	<0.001
NO_3_^–^	+0.100	0.331	+0.308	0.072
Br^–^	NA	NA	+0.318	0.084

**R, correlation coefficient; + and −, positive and negative correlation, respectively. NA, indicates not available.**

### Variations in Microbial Community Compositions After Sediment Transplantation

In the sediments of the incubating lakes (expect CKL_S) collected in September and November, the microbial communities were predominately composed of *Proteobacteria*, *Bacteroidetes*, *Chloroflexi*, *Firmicutes*, and *Actinobacteria* in a decreasing order. In the CKL sediments, the dominant phyla were *Proteobacteria*, *Euryarchaeota*, *Bacteroidetes*, *Actinobacteria*, and *Firmicutes* in September, and were *Proteobacteria*, *Bacteroidetes*, and *Actinobacteria* in November. In the transplanted sediments, microbial communities were dominated by *Proteobacteria*, *Firmicutes*, *Bacteroidetes*, and *Chloroflexi* in experiment 1 (simulating salinization), and by *Proteobacteria*, *Firmicutes*, *Bacteroidetes*, and *Halanaerobiaeota* in experiment 2 (simulating desalinization, Supplementary Figure S3).

**FIGURE 6:**
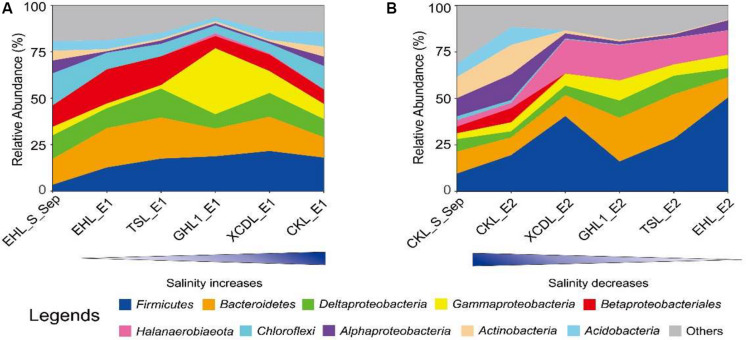
The relative abundance variation of the identified dominant taxa (at the phylum and class levels) for experiment 1 (**A**: simulating salinization) and experiment 2 (**B**: simulating desalinization).

At the end of the transplanting experiment, microbial community compositions differed greatly between the transplant sediments (incubated in different salinity lakes) and their undisturbed counterparts (Figure 6). Specifically, in experiment 1 (simulating salinization), the relative abundances of the *Chloroflexi*, *Alphaproteobacteria*, and *Actinobacteria* decreased (decreasing variation > 1%) whereas the *Firmicutes* increased in the transplanted sediments along the increasing salinity of the incubating lakes. The *Gammaproteobacteria* increased only in the transplanted sediments incubated in the (hyper)saline lakes (GHL2, XCDL, and CKL) whereas it decreased in those incubated in low salinity lakes (EHL and TSL). In contrast, the relative abundance of the *Betaproteobacteria* exerted an opposite variation along the increasing salinity of the incubating lakes (Figure 6A). In experiment 2 (simulating desalinization), the relative abundances of the *Actinobacteria* and *Acidobacteria* decreased in the transplanted sediments along the decreasing salinity of the incubating lakes, whereas the relative abundance of the *Firmicutes*, *Halanaerobiaeota*, and *Gammaproteobacteria* showed an opposite variation. The *Bacteroidetes* and *Deltaproteobacteria* were most abundant in the transplanted sediment in GHL2 and TSL (Figure 6B).

The suppressive effect of salinization and desalinization was obvious at the ASVs level. Venn diagrams demonstrated the microbial similarities (shared ASVs) and differences (unique ASVs) between the transplanted sediments (incubated in different lakes) and their undisturbed counterparts. Specifically, in experiment 1 (simulating salinization), a total of 812, 905, 813, and 973 ASVs were only identified (disappearing) in the lake sediments EHL_Sep compared with TSL, GHL2, XCDL, and CKL, respectively. In contrast, a total of 453, 284, 571, and 410 ASVs were only identified (newly appearing) in the transplanted sediments incubated in TSL, GHL2, XCDL, and CKL, respectively (Supplementary Figure S4A). Likewise, in experiment 2 (simulating desalinization), a total of 1181, 1189, 1250, and 1249 ASVs were only identified in the lake sediments CKL_Sep compared with XCDL, GHL2, TSL, and EHL, respectively. In contrast, a total of 243, 220, 197, and 163 ASVs were only identified in the transplanted sediments incubated in XCDL, GHL2, TSL, and EHL, respectively (Supplementary Figure S4B).

**FIGURE 7:**
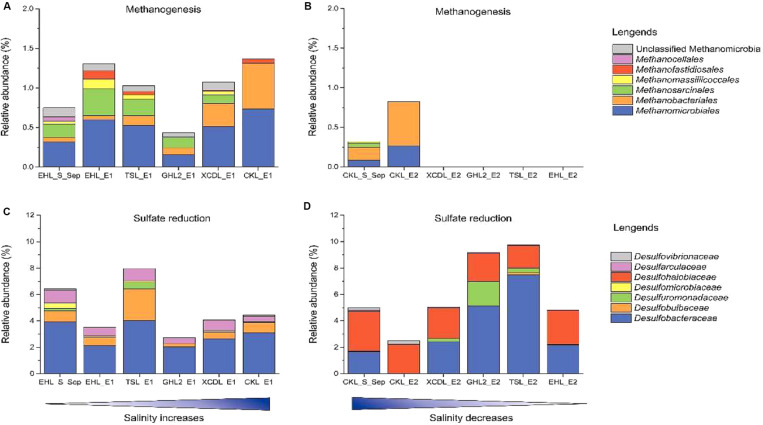
Relative abundance of methanogen and sulfate reducing taxonomic groups after sediment transplantation. **(A,C)** In experiment 1 (simulating salinization). **(B,D)** in experiment 2 (simulating desalinization).

To better understand the effect of salinization and desalinization on key functional microbial groups, taxa associated with methanogens and sulfate-reducing bacteria (SRB) were analyzed in greater detail (Figure 7). In experiment 1 (simulating salinization), microbial groups with methanogenesis potential were present in all the transplanted sediments regardless of salinity. Remarkably, the relative abundance of *Methanobacteriales* were higher in the sediments incubated in high-salinity lakes (e.g., GHL2_E1, XCDL_E1, and CKL_E1) than that in EHL (EHL_S_Sep, Figure 7A), whereas in experiment 2 (simulating desalinization), microbial groups with methanogenesis potential were only present (with low abundance) in the transplanted sediments incubated in CKL (salt-saturation), but were absent in those incubated in other lakes with lower salinity (Figure 7B). Similarly, microbial groups with sulfate reduction potential were widely distributed in all the transplanted sediments regardless of incubating lake salinity. It is notable that the taxa with sulfate reduction potential were distinct between the salinization and desalinization simulation experiments: *Desulfarculaceae*- and *Desulfobulbaceae*-related SRB were present in the transplanted sediments of experiment 1 (simulating salinization) (Figure 7C), in contrast with *Desulfohalobiaceae*-related SRB in that of experiment 2 (simulating desalinization) (Figure 7D).

### Variation in Potential Microbial Metabolic Functions in the Transplanted Sediments

**FIGURE 8:**
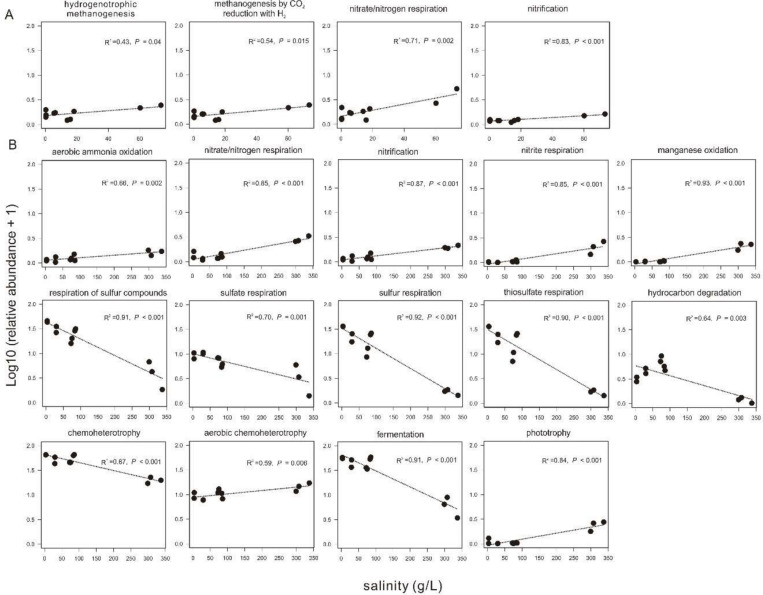
Relationship between sediment salinity and relative abundances of potential microbial functions predicted by FARPROTAX. **(A,B)** are for experiment 1 (simulating salinization) and experiment 2 (simulating desalinization), respectively.

Based on the FAPROTAX prediction analysis, 68 microbial metabolic functions (relative abundance > 0.01%) were predicted from the 16S rRNA gene sequences in all the collected sediments. In the sediments of the incubating lakes, the predicted microbial functions were similar between September and November, and mainly consisted of sulfate respiration, respiration of sulfur compounds, fermentation, and (aerobic) chemoheterotrophy. In contrast, the predicted microbial functions in the transplanted sediments at the incubation end (S_Nov) were distinct from their beginning counterpart (S_Sep) and from that in the sediment of the incubating lakes (Supplementary Figure S5). Among the predicted microbial functions in experiment 1 (simulating salinization), the relative abundance of hydrogenotrophic methanogenesis, methanogenesis through CO_2_ reduction with H_2_, nitrate/nitrogen respiration, and nitrification showed significant (*P* < 0.05) positive correlation with the salinity in the transplanted sediments (Figure 8A). Similarly, in experiment 2 (simulating desalinization), the relative abundance of respiration of sulfur compounds, sulfate respiration, sulfur respiration, thiosulfate respiration, hydrocarbon degradation, chemoheterotrophy, and fermentation was significantly (*P* < 0.01), negatively correlated with the salinity in the transplanted sediments, whereas the relative abundance of aerobic ammonia oxidation, nitrate/nitrogen respiration, nitrification, nitrite respiration, manganese oxidation, aerobic chemoheterotrophy, and phototrophy displayed a significantly (*P* < 0.01) positive correlation with the salinity in the transplanted sediments (Figure 8B).

## Discussion

The desalinization process in the transplanted sediments was faster than salinization. After 50-day incubation, the salinity of the transplanted sediments in the desalinization simulation experiment was in equilibrium with the incubating lakes; however, such an equilibrium was not observed for the salinity in the salinization simulation experiment. This finding could be ascribed to the following fact: in the salinization simulation experiments, osmotic stress resulted in salt-in and water-out, and thus dry microenvironment was formed in the caged sediments, which did not avail to further exchange water and salt between inside and outside of the cages; whereas an opposite water–salt exchange trend (i.e., water-in and salt-out) took place in the desalinization simulation experiments, and water–salt exchange became easier as desalinization proceeded (Brown, 1976, 1990; Oren, 2008). So it is not surprising to observe the different velocity for desalinization and salinization.

Exposure to the hypersaline or freshwater environment changed the geochemistry and salinity of porewater in the transplanted sediments, suggesting that the transplantation was able to mimic the effect of salinization and desalinization. For example, the salinity of the transplanted sediments increased from 0.3 to 73.4 g/L (maximum) in the salinization simulation experiments, whereas the salinity in the transplanted sediments decreased from 299.3 to 3.2 g/L (minimum) in the desalinization simulation experiments (Figure 1). Such salinity change was expected and was consistent with previous transplanting studies (Reed and Martiny, 2013; Huang et al., 2019). Furthermore, the TOC contents in the transplanted sediments incubated in the lakes with high salinity were lower than in their counterparts with low salinity in experiment 1, and TOC in the transplanted sediments significantly increased with decreasing salinity (*P* < 0.01) in experiment 2 (Supplementary Figure S2). Such a relationship between salinity and TOC contents was ever observed previously (Wolaver et al., 1986; Craft et al., 1989). This could be explained by the fact that salinity can affect organic carbon lability and sorption dynamics that can influence the availability of carbon substrates for microbial metabolism (Mavi and Marschner, 2017). A large quantity of carbon substrates in freshwater environments may be important for microorganisms to alleviate some adverse effects of salinity (Dang et al., 2019). The changes in physicochemical properties may account for the variations of microbial community compositions and predicted microbial functions in the transplanted sediments. However, the relationship between salinity and TOC contents was not observed in the sediments of the five incubating lakes. This could be ascribed to the fact that the TOC contents mainly depend on lake sedimentary characteristics (e.g., plant debris, mineralogy) rather than salinity. In addition, salinity is often related to other environmental stresses, such as water content and pH (Rath and Rousk, 2015), which together with other geochemical parameters (e.g., temperature, mineralogy) were also expected to take effect in shaping microbial communities in the salinization and desalinization simulation experiments (Wang et al., 2014; Yang et al., 2020). This point still awaits further investigation.

It is notable that microbial diversity in the transplanted sediments was lower than that in the sediments of the incubating lakes in both experiments. Microbial diversity decreased in response to salinization, which was understandable in that microbial diversity decreased with increasing salinity (Zhang K. et al., 2019). In contrast, microbial diversity also decreased in response to desalinization, which may be ascribed to the fact that some microbes in the transplanted CKL sediments cannot grow at low salinity of the incubating lakes. One previous study showed that many halophilic microorganisms would die of low osmosis (Kunte et al., 2002), leading to microbial diversity reduction. So it is reasonable to observe microbial diversity decreased in response to both salinization and desalinization.

Different microbial components showed different variations in response to salinization and desalinization. For example, in the salinization simulation experiments, the relative abundance of *Firmicutes* and *Gammaproteobacteria* increased whereas *Betaproteobacteria* and *Chloroflexi* decreased in the transplanted sediments incubated in the lakes with high salinity (Figure 6A); in the desalinization simulation experiments, the relative abundance of *Actinobacteria* decreased whereas *Firmicutes*, *Halanaerobiaeota*, and *Bacteroidetes* increased in the transplanted sediments incubated in the lakes with low salinity (Figure 6B). Previous studies found that the *Gammaproteobacteria* and *Actinobacteria* were prevalent in saline environments (Wu et al., 2006; Chen et al., 2017; Liu et al., 2018) whereas the *Betaproteobacteria*, *Bacteroidetes*, and *Chloroflexi* became abundant in low-salinity environments (Zwart et al., 2002; Barberán and Casamayor, 2010; Mehrshad et al., 2018; Kim et al., 2019). Our observations are in line with these studies. Such microbial variations could be ascribed to the effect of salt increase or decrease on microbes. In response to salinization or desalinization, microbes have to cope with increased or decreased osmotic pressure by changing their physiology (Killham, 1994) and/or morphology (Zahran, 1997). Two adaptation strategies could be used by microorganisms to cope with osmotic stress: one is the “salt-in” strategy, which involves taking up molar concentrations of potassium and chloride, and the other is to accumulate organic solutes (e.g., amino acids, carbohydrates) within cells (Oren, 2008). Different microorganisms prefer different adaptation strategies. Therefore, it is reasonable to observe the microbial variations in response to salinization and desalinization. In addition, it is interesting to observe the *Halanaerobiaeota* became more abundant in the sediments with low salinity (Figure 6B). To our knowledge, the *Halanaerobiaeota* consists of anaerobic fermentative bacteria that are halotolerant to halophilic (Roush et al., 2014), and some *Halanaerobiaeota* species require relatively low salt for growth (Oren, 2008) and may have efficient strategies to adapt to low salinity (Yang et al., 2018). Furthermore, some halophilic microorganisms have a remarkable ability to withstand low salt conditions (Torreblanca et al., 1986; Fukushima et al., 2007) and even retain their viability in distilled water (Savage et al., 2007). Therefore, it is reasonable to observe the predominance of *Halanaerobiaeota* in the transplanted sediments incubated at low salinity.

Considering that the dialysis bags employed in the transplanting experiments could prevent microbes from exchanging between inside and outside of the established cages, the effect of salinization and desalinization was also obvious at the ASV level in the transplanted sediments. For example, some microbial species disappeared whereas others appeared at the incubating conditions. One possible reason for this phenomenon is that some rare or dormant taxa (serve as “seed bank”) existing in the beginning lake sediments evaded detection (Sjöstedt et al., 2012) but may become abundant in the corresponding transplanted sediments incubated in another lake with different salinity. This finding is consistent with the putative rationale of “everything is everywhere, but the environment selects” (De Wit and Bouvier, 2006). Another reason for the variation of certain microbial groups could be ascribed to the intrinsic limitations (e.g., PCR amplification randomness and primer selectivity) of the PCR-based high-throughput sequencing (Kircher and Kelso, 2010).

Different metabolic functions responded differently to the salinization and desalinization effects. This could be ascribed to the fact that extracellular osmolarity of microbial cells increases in response to enhanced salinity, which costs more energy than growth at the beginning salinity (Oren, 2011; Emmerich et al., 2012; Rath and Rousk, 2015). Therefore, desalinization would enhance some metabolic functions (e.g., sulfur respiration, hydrocarbon degradation in this study). Such phenomenon was consistent with previous studies. For example, the genera of *Halomonos*, *Pseudoaltermonas*, and *Vibrio* within the *Gammaproteobacteria* in low-salinity lakes showed stronger carbon utilization ability than their counterparts from high-salinity lakes (Liu et al., 2018). Note that some microbial metabolic functions still remained active at high salinity, such as fermentation, aerobic chemoheterotrophy, methanogenesis-related functions, nitrate reduction, respiration of sulfur compounds (e.g., sulfate reduction), and iron respiration, which were all dissimilatory metabolisms. This finding could be explained by the fact that some microorganisms could adapt to high osmotic stress by generating enough energy during their dissimilatory metabolisms (Oren, 2011). For example, denitrification and microbial iron cycle could occur at salinity close to the solubility limit of NaCl (Mancinelli and Hochstein, 1986; Oren, 2011; Emmerich et al., 2012). Methanogenesis through CO_2_ reduction with H_2_ and through acetate were ever found at salt concentrations up to 100 g/L (Oren, 2011), and methanogenesis with the use of methylated amines, methanol, and dimethylsulfide became predominant at higher salt concentrations (McGenity, 2010). Furthermore, microbial sulfate reduction happened in a wide range of salinity up to salt saturation (Oren, 2011) and became abundant in low-salinity environment with high TOC (Weston et al., 2011). Therefore, it is reasonable to observe the variations of the predicted metabolic functions with salinity in the transplanting experiments simulating salinization and desalinization.

Transplantation manipulation and salinity are two major factors structuring microbial community development in both the salinization and desalinization simulation experiments. On one hand, transplantation operation could contribute to the observed microbial difference between the transplanted sediments and their undisturbed counterparts. This finding could be ascribed to the two following reasons: (1) microbial communities could change with time, which could be evidenced by the microbial difference between the incubating lake sediments sampled in September and November; and (2) anthropogenic perturbations (e.g., mixing lake sediments before caging, caging the lake sediments into the dialysis bags) would change the redox conditions of microenvironments in the caged sediments, leading to a shift of the microbial community compositions. So it is not surprising to observe that the microbial communities in the transplanted sediments retrieved from both EHL_E1 and CKL_E2 were different from that in their undisturbed counterparts. Given that incubation time may affect the microbial community structure (Pernthaler et al., 1998; Zhang Y. et al., 2017), it is interesting to note that the community compositions in the transplanted sediments were also distinct from that in the incubating lake sediment sampled in November. However, the microbial community compositions of the transplanted sediments could not reflect a gradient community with undisturbed incubating lakes. In this study, transplantation experiments were established by caging the lake sediments (the freshwater lake end EHL_S_Sep or the salt lake end CKL_S_Sep) into semipermeable membrane, which prevented microbes from exchanging between inside and outside. This means that the microbial community compositions in the transplanted sediments could only be influenced by variation of physicochemical factors (e.g., salinity) caused by transplantation. The resulting change in salinity stress may have selected for taxa capable of maintaining activity at the newly established physicochemical conditions in the salinization and desalinization simulation experiments. Hence, only microbes capable of adapting to higher salinity in experiment 1 (simulating salinization) or lower salinity in experiment 2 (simulating desalinization) could survive. Therefore, in response to (de)salinization, microbial succession may not proceed on a direct path from freshwater to hypersaline environments or the other way round, and instead (de)salinization may lead to unique transitional communities characteristic of taxa uncommon in both freshwater and hypersaline environments (Dang et al., 2019).

On the other hand, salinity accounted for the observed difference among the samples, and the greater the salinity difference, the greater the community difference. Specifically, in experiment 1 (simulating salinization), the salinity in the caged sediments were not fully equilibrated with (or equal to) their new incubating lakes after 50-day incubation (Supplementary Table S1). The salinity of the transplanted sediments TSL_E1 (salinity, 5.9 g/L), GHL2_E1 (salinity, 14.9 g/L), and XCDL_E1 (salinity, 18.2 g/L) was lower than that of seawater (35 g/L). Most if not all microbes in such sediments would be capable of tolerating salinity up to 35 g/L, and thus small salinity variation (from freshwater to seawater salinity) may not significantly alter the microbial community compositions in the transplanted sediments (Edmonds et al., 2009). Therefore, it is reasonable to observe the grouping of the microbial communities of the transplanted sediments EHL_E1, TSL_E1, GHL2_E1, and XCDL_E1. In contrast, the salt concentrations of CKL_E1 (salinity, 60.2–73.4 g/L) was higher than twice that of seawater, and was beyond the salinity that most microbes can tolerate. Thus, the microbial communities in the transplanted sediments CKL_E1 were clearly segregated from that in EHL_S_Sep (Figure 4A). In experiment 2 (simulating desalinization), the salinity in the caged sediments was almost in equilibrium with that of the incubating lakes (Supplementary Table S1). Although transplantation manipulation might change the microbial community compositions in the transplanted sediments, the communities of CKL_E2 were more closely clustered with CKL_S_Sep than with other transplanted sediments (Figure 4B). Because of the nature of high salt concentration (salinity, 299.3 g/L), the CKL_S_Sep sediments comprised a variety of extremely halophilic and halotolerant microorganisms. Previous studies showed that extreme halophiles and halotolerants required salinity of 200–300 g/L for optimum growth but could not grow at salinity lower than 100 g/L (Kunte et al., 2002; Kerkar, 2004). In the present study, the salinity of the transplanted sediments XCDL_E2, GHL2_E2, TSL_E2, and EHL_E2 was lower than 100 g/L, and was far lower than their undisturbed counterparts CKL_S_Sep (salinity, 299.3 g/L). Therefore, most if not all extreme halophiles and halotolerants could not withstand low salinity and thus die of low osmosis (Kunte et al., 2002). So it is not surprising to observe the tight grouping of these caged sediments. Therefore, taken together, salinity might play more important roles than transplantation manipulation in shifting the microbial community compositions from the undisturbed sediments to their transplanted counterparts.

## Conclusion

In conclusion, salinization and desalinization had strong effects on sedimentary geochemistry, microbial community compositions, and functions in the transplanted sediments, and the desalinization process was faster than salinization. In response to salinization and desalinization, microbial diversity decreased and microbial taxonomic and functional compositions succeeded with the emergence of a transitional microbial community. The variations in microbial community compositions and predicted functions are likely dependent on integration of transplantation, salinity, and covarying variables. Microbial communities might adapt to salinization and desalinization by regulating the relative abundance and functions of certain taxa. Taken together, this study expands our current knowledge about microbial response to salinization or desalinization in lakes with salinity from freshwater to salt saturation.

## Data Availability Statement

The datasets presented in this study can be found in online repositories. The names of the repository/repositories and accession number(s) can be found in the article/ Supplementary Material.

## Author Contributions

HJ conceived the study. JY, HJ, and GW collected the samples and performed on-site simulation experiments. JHu, WL, BW, HX, and JHa analyzed geochemistry and microbiology of the samples. JHu analyzed the sequencing data. HJ and JHu drafted the article. All authors reviewed results and commented on the article.

## Conflict of Interest

The authors declare that the research was conducted in the absence of any commercial or financial relationships that could be construed as a potential conflict of interest.

## Footnotes

1 https://qiime2.org/

2 http://cran.r-project.org/
