# Supplementary material for: Microbial Responses to Simulated Salinization and Desalinization in the Sediments of the Qinghai–Tibetan Lakes
Source: Front Microbiol. 2020 Aug 7;11:1772. doi: 10.3389/fmicb.2020.01772 (PMC7426462; doi:10.3389/fmicb.2020.01772)
Supplement: Supplementary file 1 [file Data_Sheet_1.DOCX]

**SUPPORTING INFORMATION**

**Supplementary Table S1** Environmental variables in the studied samples.

| Sample IDs | Porewater salinity (g/L) | Porewater pH | TOC (%) | K^+^ (mg/L) | Na^+^ (mg/L) | Ca^2+^ (mg/L) | Mg^2+^ (mg/L) | Li^+^ (mg/L) | Cl^-^ (mg/L) | SO_4_^2-^ (mg/L) | NO_2_^-^ (mg/L) | NO_3_^-^ (mg/L) | Br^-^ (mg/L) |
| --- | --- | --- | --- | --- | --- | --- | --- | --- | --- | --- | --- | --- | --- |
| EHL_S_Sep | 0.3 | 8.2 | 3.4 | 26.3 | 62.0 | 27.4 | 59.4 | 0.1 | 113.2 | 46.8 | 11.5 | 0.0 | 0.0 |
| TSL_S_Sep | 25.3 | 8.1 | 1.3 | 254.7 | 3466.8 | 1806.8 | 152.0 | 1.4 | 12425.6 | 7098.0 | 52.3 | 0.4 | 0.0 |
| GHL2_S_Sep | 33.5 | 8.2 | 4.3 | 582.8 | 5705.8 | 1837.7 | 226.7 | 7.0 | 19688.6 | 5304.7 | 124.3 | 0.4 | 0.0 |
| XCDL_S_Sep | 75.2 | 8.6 | 1.0 | 580.6 | 14172.6 | 2171.3 | 350.3 | 11.4 | 41528.8 | 15406.2 | 169.5 | 0.0 | 828.5 |
| CKL_S_Sep | 299.3 | 7.3 | 0.6 | 5221.1 | 32888.5 | 32812.9 | 267.3 | 17.9 | 181890.9 | 45936.7 | 297.7 | 0.4 | 0.0 |
| EHL_S_Nov | 0.8 | 8.5 | 0.7 | 26.4 | 199.3 | 96.5 | 18.8 | 0.2 | 304.2 | 93.7 | 19.9 | 0.0 | 9.5 |
| TSL_S_Nov | 25.4 | 8.2 | 0.3 | 232.8 | 5075.9 | 1783.4 | 95.5 | 0.0 | 11484.4 | 6618.0 | 58.9 | 60.4 | 0.0 |
| GHL2_S_Nov | 29.3 | 7.8 | 3.7 | 549.8 | 6861.2 | 1423.4 | 463.5 | 0.0 | 15774.2 | 4102.6 | 155.1 | 0.0 | 0.0 |
| XCDL_S_Nov | 78.1 | 8.1 | 0.2 | 576.9 | 20417.7 | 1825.9 | 487.6 | 0.0 | 40164.5 | 14418.2 | 235.9 | 0.0 | 0.0 |
| CKL_S_Nov | 350.7 | 7.7 | 1.6 | 6210.1 | 55366.4 | 37291.8 | 376.6 | 12.7 | 208324.4 | 41240.9 | 389.8 | 558.9 | 911.4 |
| EHL_E1_1 | 0.4 | 8.2 | 2.6 | 9.4 | 64.2 | 22.5 | 134.3 | 0.0 | 66.4 | 42.3 | 12.9 | 5.5 | 0.0 |
| EHL_E1_2 | 0.3 | 8.3 | 2.2 | 12.1 | 64.8 | 24.7 | 62.5 | 0.0 | 67.9 | 60.5 | 12.4 | 0.0 | 0.0 |
| TSL_E1_1 | 5.5 | 8.4 | 1.9 | 77.9 | 1234.7 | 331.6 | 0.0 | 0.0 | 2549.5 | 1313.5 | 32.7 | 0.0 | 0.0 |
| TSL_E1_2 | 6.3 | 8.3 | 2.0 | 79.1 | 1379.4 | 383.4 | 18.8 | 0.0 | 2905.9 | 1474.7 | 31.8 | 0.0 | 0.0 |
| GHL2_E1_1 | 13.9 | 7.8 | 1.7 | 240.9 | 2801.1 | 597.4 | 99.2 | 0.0 | 7257.4 | 2281.9 | 95.9 | 558.8 | 0.0 |
| GHL2_E1_2 | 15.9 | 7.7 | 2.1 | 161.3 | 3266.9 | 729.4 | 111.6 | 0.0 | 8468.6 | 2510.5 | 112.1 | 566.8 | 0.0 |
| XCDL_E1_2 | 18.2 | 8.1 | 2.0 | 190.7 | 4324.3 | 338.0 | 68.9 | 0.0 | 8701.5 | 3791.9 | 178.0 | 586.8 | 0.0 |
| CKL_E1_1 | 73.4 | 7.8 | 2.0 | 1424.8 | 10736.3 | 8427.9 | 0.0 | 0.0 | 43931.2 | 8664.0 | 186.5 | 0.0 | 0.0 |
| CKL_E1_2 | 60.2 | 7.8 | 2.2 | 1171.4 | 8778.8 | 6901.9 | 271.9 | 0.0 | 35796.0 | 7177.4 | 123.2 | 0.0 | 0.0 |
| EHL_E2_1 | 3.5 | 7.9 | 2.1 | 39.2 | 226.8 | 186.6 | 548.9 | 0.3 | 328.1 | 2185.8 | 7.8 | 0.0 | 11.0 |
| EHL_E2_2 | 3.2 | 7.9 | 1.2 | 27.7 | 213.0 | 170.4 | 511.9 | 0.2 | 292.6 | 1975.1 | 11.1 | 5.6 | 11.5 |
| TSL_E2_1 | 29.8 | 8.1 | 1.7 | 293.7 | 5380.8 | 2011.7 | 624.8 | 0.0 | 11921.2 | 9449.9 | 64.8 | 0.0 | 94.7 |
| TSL_E2_2 | 29.4 | 7.8 | 1.9 | 302.9 | 5245.2 | 2046.5 | 614.5 | 0.0 | 11642.3 | 9270.9 | 74.8 | 66.5 | 102.6 |
| GHL2_E2_1 | 75.2 | 8.1 | 0.9 | 538.0 | 16792.7 | 3634.3 | 842.7 | 0.0 | 40095.2 | 12557.2 | 120.8 | 612.7 | 0.0 |
| GHL2_E2_2 | 72.5 | 8.1 | 0.7 | 477.6 | 16373.0 | 3486.0 | 838.3 | 0.0 | 39117.8 | 12062.5 | 180.7 | 0.0 | 0.0 |
| XCDL_E2_1 | 83.4 | 8.3 | 1.7 | 676.6 | 21403.4 | 1884.3 | 694.3 | 0.0 | 40825.4 | 17661.4 | 220.8 | 0.0 | 0.0 |
| XCDL_E2_2 | 85.7 | 8.5 | 1.3 | 676.7 | 22001.7 | 1862.5 | 700.7 | 0.0 | 41834.6 | 18393.7 | 247.6 | 0.0 | 0.0 |
| CKL_E2_1 | 307.7 | 7.6 | 0.6 | 5642.2 | 48079.4 | 33326.2 | 391.6 | 9.8 | 182844.0 | 36468.9 | 342.7 | 579.4 | 0.0 |
| CKL_E2_2 | 337.6 | 7.8 | 0.4 | 6273.6 | 52343.1 | 36674.0 | 480.6 | 11.1 | 201007.0 | 38966.9 | 404.5 | 562.6 | 925.7 |

Note: TOC: total organic carbon; S represents sediment; E1 and E2 represent the field experiment 1 (simulating salinization) and field experiment 2 (simulating desalinization), respectively; Sep and Nov indicate the sampling time in September and November, respectively.

**Supplementary Table S2** The diversity indices of the investigated samples based on the rarefied sequence data (*n*=5744).

| Sample IDs | Number of  ASVs | Shannon  index | Simpson | Equitability | Fisher.alpha |
| --- | --- | --- | --- | --- | --- |
| EHL_S_Sep | 1085 | 6.11 | 0.99 | 0.87 | 395.72 |
| TSL_S_Sep | 820 | 5.72 | 0.99 | 0.85 | 261.71 |
| GHL2_S_Sep | 823 | 5.58 | 0.99 | 0.83 | 263.09 |
| XCDL_S_Sep | 754 | 5.38 | 0.99 | 0.81 | 232.12 |
| CKL_S_Sep | 1409 | 6.38 | 0.99 | 0.88 | 595.88 |
| EHL_S_Nov | 898 | 5.86 | 0.99 | 0.86 | 298.58 |
| TSL_S_Nov | 684 | 5.22 | 0.98 | 0.80 | 202.33 |
| GHL2_S_Nov | 673 | 4.69 | 0.94 | 0.72 | 197.79 |
| XCDL_S_Nov | 522 | 4.81 | 0.98 | 0.77 | 139.50 |
| CKL_S_Nov | 564 | 4.85 | 0.98 | 0.77 | 154.98 |
| EHL_E1_1 | 773 | 5.38 | 0.98 | 0.81 | 240.49 |
| EHL_E1_2 | 794 | 5.51 | 0.98 | 0.83 | 249.88 |
| TSL_E1_1 | 690 | 5.12 | 0.97 | 0.78 | 204.82 |
| TSL_E1_2 | 720 | 5.28 | 0.98 | 0.80 | 217.45 |
| GHL2_E1_1 | 450 | 3.64 | 0.91 | 0.60 | 114.31 |
| GHL2_E1_2 | 480 | 4.60 | 0.95 | 0.75 | 124.60 |
| XCDL_E1_2 | 892 | 5.69 | 0.99 | 0.84 | 295.67 |
| CKL_E1_1 | 563 | 5.53 | 0.99 | 0.87 | 154.60 |
| CKL_E1_2 | 570 | 5.55 | 0.99 | 0.87 | 157.23 |
| EHL_E2_1 | 281 | 3.67 | 0.91 | 0.65 | 61.87 |
| EHL_E2_2 | 266 | 3.62 | 0.90 | 0.65 | 57.69 |
| TSL_E2_1 | 301 | 3.98 | 0.95 | 0.70 | 67.57 |
| TSL_E2_2 | 274 | 3.92 | 0.95 | 0.70 | 59.91 |
| GHL2_E2_1 | 345 | 4.18 | 0.96 | 0.71 | 80.60 |
| GHL2_E2_2 | 367 | 4.13 | 0.95 | 0.70 | 87.36 |
| XCDL_E2_1 | 401 | 3.85 | 0.93 | 0.64 | 98.13 |
| XCDL_E2_2 | 354 | 3.54 | 0.91 | 0.60 | 83.35 |
| CKL_E2_1 | 789 | 5.75 | 0.99 | 0.86 | 247.63 |
| CKL_E2_2 | 734 | 5.66 | 0.99 | 0.86 | 223.45 |

Note: amplicon sequence variants (ASVs) were classified using a 100% nucleotide sequence identity cutoff.

**Supplementary Figure S1** Geographic map showing the sampling sites on the Qinghai-Tibetan Plateau in the study. Abbreviations: EHL: Erhai Lake; TSL: Tuosu Lake; GHL2: Gahai Lake 2; XCDL: Xiaochaidan Lake; CKL: Chaka Lake.

**
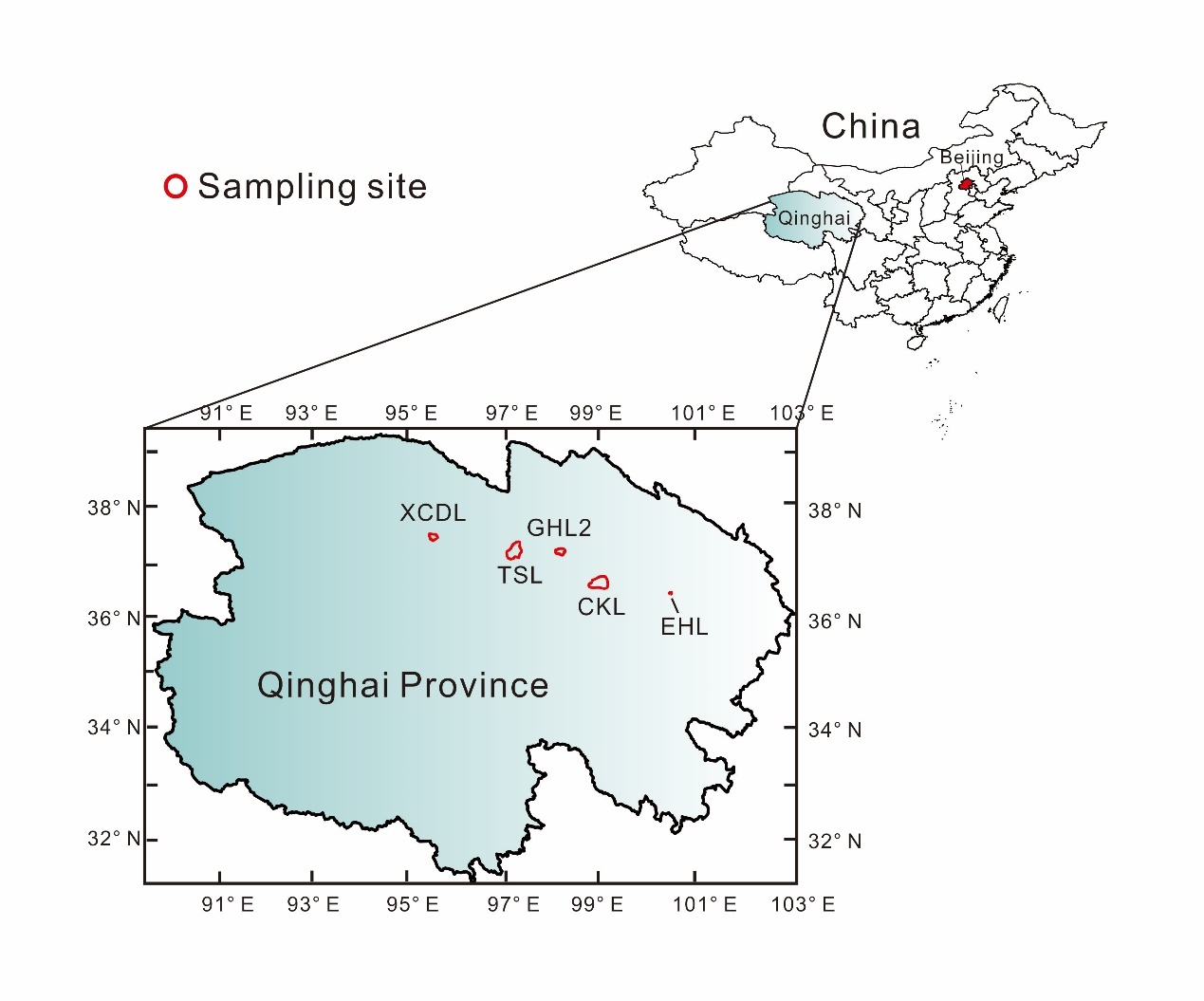
**

**Supplementary Figure S2** Linear regression analysis between salinity and TOC contents in the transplanted sediments. Panels A and B are for the experiment 1 (simulating salinization) and experiment 2 (simulating desalinization), respectively.

**
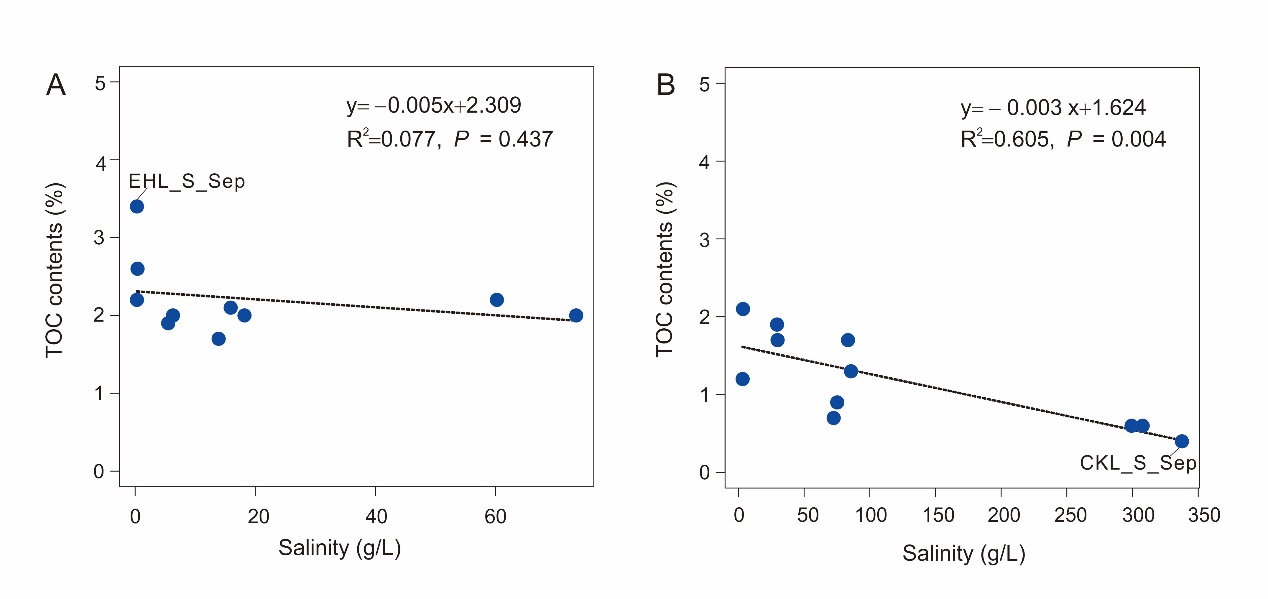
**

**Supplementary Figure S3** Microbial community compositions at the phylum level (>1% relative abundance) in the transplanted and incubating lake sediments. Only top 10 abundant phylogenetic phyla/classes were shown. Panel A: field experiment 1 (simulating salinization); Panel B: field experiment 2 (simulating desalinization); Panel C: sediments of incubating lakes collected in September; Panel D: sediments of incubating lakes collected in November. In the field experiment 1, the transplanted EHL sediments caged in XCDL (XCDL_E1_1) was not successfully retrieved and thus was removed in the figure. Note: Sep., September; Nov., November.

**
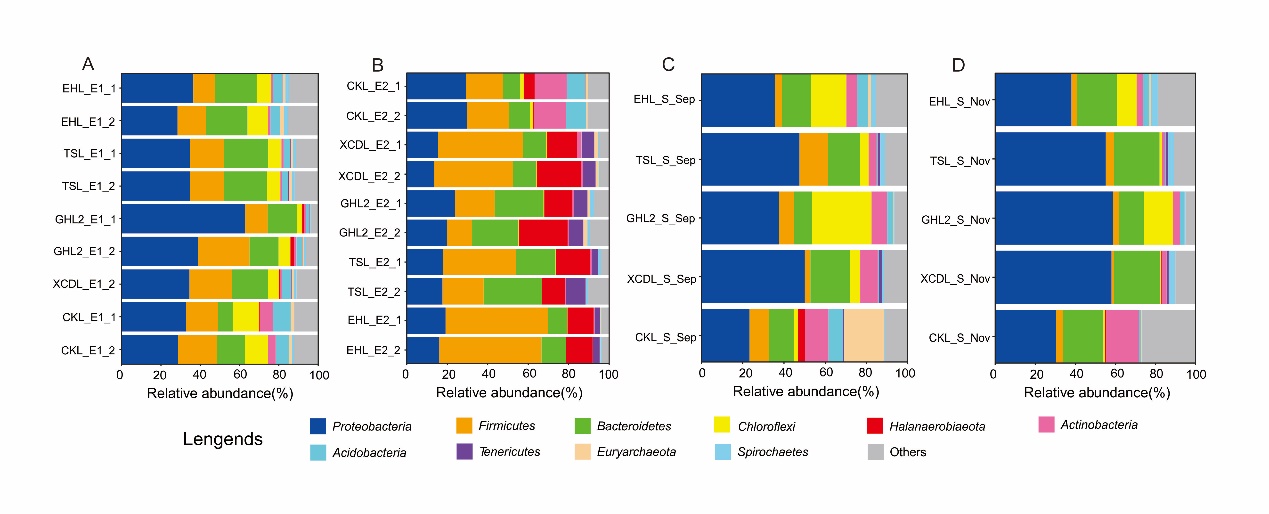
**

**Supplementary Figure S4** Venn diagram illustrating the microbial similarity (shared amplicon sequence variants, abbreviated as ASVs) and difference (unique ASVs) between the transplanted sediments and their undisturbed counterparts in the field experiment 1 (A panel: simulating salinization) and field experiment 2 (B panel: simulating desalinization).

**
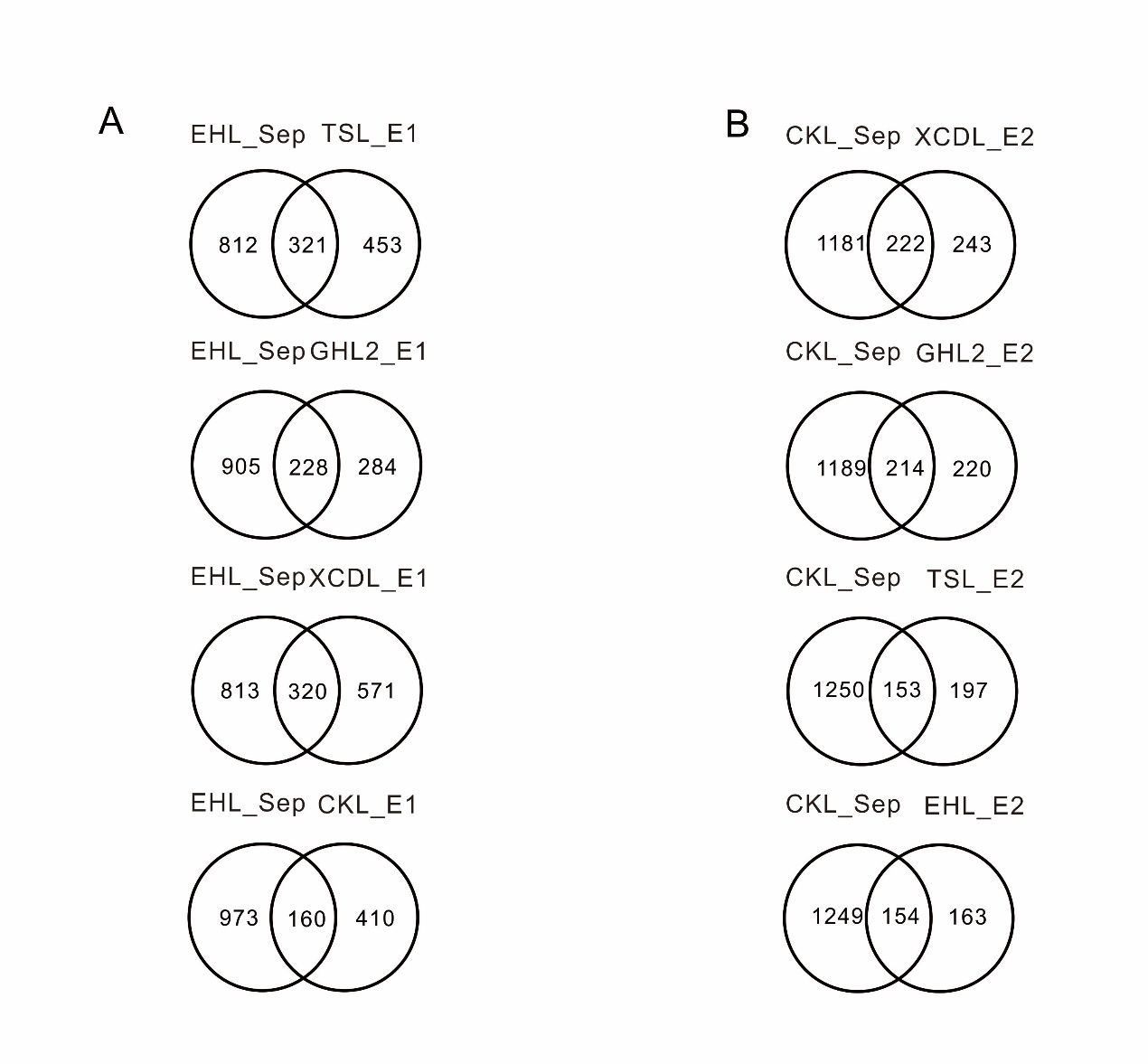
**

**Supplementary Figure** **S5** The relative abundance of the abundant predicted metabolic functions in the lake and transplant sediments. Panel A: field experiment 1 (simulating salinization); Panel B: field experiment 2 (simulating desalinization); Panel C: sediments of incubating lakes sampled in September; Panel D: sediments of incubating lake sampled in November. In the field experiment 1, the transplanted EHL sediments caged in XCDL (XCDL_E1_1) was not successfully retrieved and thus was removed in the figure. Note: Sep., September; Nov., November..

**
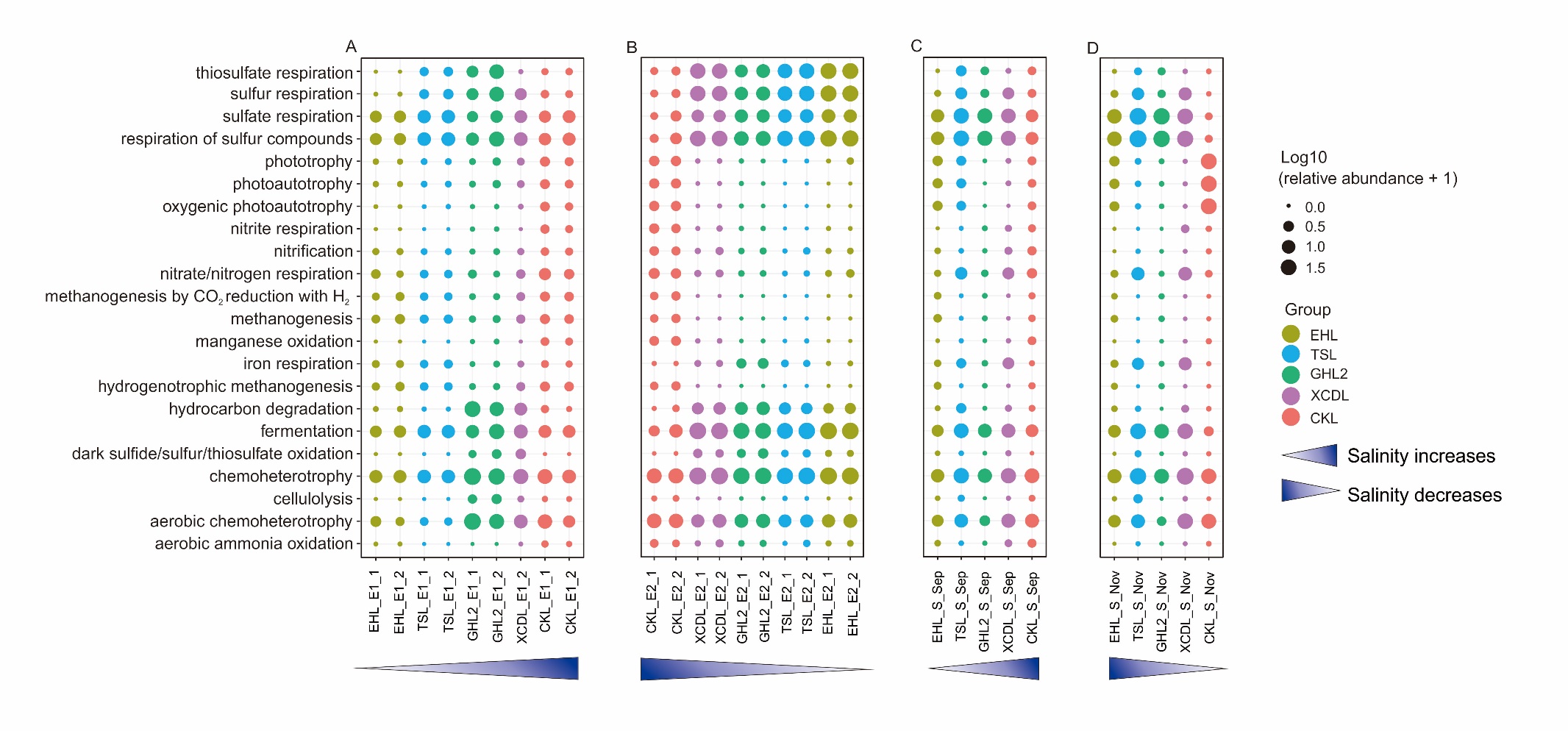
**
